# Soil nutrients and plant diversity affect ectomycorrhizal fungal community structure and functional traits across three subalpine coniferous forests

**DOI:** 10.3389/fmicb.2022.1016610

**Published:** 2022-10-06

**Authors:** Nan Yang, Jiani Hua, Jiangbao Zhang, Dong Liu, Parag Bhople, Xiuxiu Li, Yan Zhang, Honghua Ruan, Wei Xing, Lingfeng Mao

**Affiliations:** ^1^Department of Ecology, College of Biology and the Environment, Co-Innovation Center for Sustainable Forestry in Southern China, Nanjing Forestry University, Nanjing, China; ^2^The Germplasm Bank of Wild Species, Yunnan Key Laboratory for Fungal Diversity and Green Development, Kunming Institute of Botany, Chinese Academy of Sciences, Kunming, Yunnan, China; ^3^Department of Biological Sciences, Faculty of Science and Engineering, School of Natural Sciences, University of Limerick, Limerick, Ireland; ^4^Jiangsu Academy of Forestry, Nanjing, China; ^5^Yangzhou Urban Forest Ecosystem National Research Station, Jiangsu, Yangzhou, China

**Keywords:** ectomycorrhizal fungi, subalpine region, fungal ITS gene, coniferous forest ecosystems, climate warming

## Abstract

The symbiotic relationship between ectomycorrhizal fungi (EMF) and the roots of host plants is significantly important in regulating the health and stability of ecosystems, especially of those such as the climate warming affected subalpine forest ecosystems. Therefore, from the coniferous forest systems located in the Southern Qinghai-Tibetan Plateau, root tips from three forest tree species: *Pinus wallichiana*, *Abies spectabilis and Picea spinulosa*, were collected to look for the local causes of EMF community composition and diversity patterns. The EMF colonization rate, diversity and taxonomic community structure were determined by morphotyping and sanger sequencing of the fungal ITS gene from the root tip samples. Soil exploration types were identified based on the morphologies of the ectomycorrhizas, coupled with soil properties analysis and plant diversity survey. Contrasting patterns of EMF community and functional diversity were found across the studied three forests types dominated by different coniferous tree species. In terms of associations between soil and EMF properties, the total phosphorus (TP) and nitrate (NO_3_^−^) contents in soil negatively correlated with the colonization rate and the Shannon diversity index of EMF in contrast to the positive relationship between TP and EMF richness. The soil total nitrogen (TN), ammonium (NH_4_^+^) and plant diversity together caused 57.6% of the total variations in the EMF taxonomic community structure at the three investigated forest systems. Whereas based on the soil exploration types alone, NH_4_^+^ and TN explained 74.2% of variance in the EMF community structures. Overall, the findings of this study leverage our understanding of EMF dynamics and local influencing factors in coniferous forests dominated by different tree species within the subalpine climatic zone.

## Introduction

The impacts of climate change on forest processes are driven by disturbances in biogeochemical cycling and decline in soil biodiversity (van der Linde et al., 2018). The coniferous forests are important characteristics of subalpine region which are currently experiencing the adverse effects of environmental stress such as the rise in temperature (Wang et al., 2021a). In forest ecology, ectomycorrhizal fungi (EMF) are an interface between the soil matrix and the host tree species. They often regulate the carbon and nutrients balance (Lindahl and Tunlid, 2015; Lilleskov et al., 2019), and therefore are of significant importance in network building and maintaining the health and stability of the forest ecosystem (Nash et al., 2020; Vincent and Declerck, 2021). In turn, the various biotic (plants and other organisms) and abiotic factors (e.g., the soil pH and niche specific microclimatic conditions) within the forest ecosystem affect the dynamics of EMF communities (Roy et al., 2013; Pei et al., 2016; Glassman et al., 2017). In such scenario, understanding more about the EMF community ecology and diversity would help us to prevision the response of forest ecosystems to changing climatic conditions, especially in the globally important subalpine-cold Himalayan regions (Shi et al., 2018; Shigyo and Hirao, 2021).

The photosynthetically derived carbon (C) from host plants is critical for EMF who in return, acquire nutrients for host plants by directly emanating their hyphae to scavenge inorganic nutrients, or through indirect provisioning of organic nutrients *via* enzymatic decomposition of the organic matter in soils (Bogar et al., 2022). The EMF and host plant species are therefore highly specific and any changes in diversity of host plant species will greatly affect the foraging-related functional traits of the symbiotic EMF community (Reis et al., 2018). Seemingly, the higher plant diversity increases diverse food resources availability and diversity through differential litter input and/or root exudates, and elevates niche availability through changes in physical microhabitats (Dedeyn and Vanderputten, 2005). Such changes in plant diversity were related to positive effects and increase in the diversity in EMF community composition (Yang et al., 2022). Whereas, other studies found no direct relationships between plant and EMF diversity, and that plant diversity only influenced the occurrence of few particular fungi (Arraiano-Castilho et al., 2020; Adamo et al., 2021). Furthermore, a few studies indicated EMF community filtering through host plant specificity (Tedersoo et al., 2012) and, increase in EMF diversity directly related to increase in host tree diversity (Gao et al., 2013). Contrasting to this, the EMF diversity was highest in temperate and boreal forests despite of the low diversity of host tree species (Rosinger et al., 2018). These inconsistent observations in the plant-EMF interactions might as well be attributed to altering soil properties (including soil pH and nutrients) in different forest ecosystems thereby indicating a more complex mechanism that regulates the dynamics of EMF community composition and diversity patterns.

Increasing local-scale studies suggested soil properties as the dominant factors shaping EMF communities across various ecosystems (Taylor et al., 2016; Arraiano-Castilho et al., 2021). In European oak forests, along with N deposition, soil conditions and properties (increasing N availability and decreasing pH) significantly affected EMF diversity (Suz et al., 2014). Among such soil properties, soil pH was the confounding regulator of EMF communities and thereby regarded as key environmental filter for divergence in the EMF taxa (Glassman et al., 2017; Tedersoo et al., 2020). Apparently, soil pH also modulated mobility of soil nutrients such as the Nitrogen (N) and Phosphorus (P) (Smith and Read, 2008), and any variations in their contents reflected changes in EMF communities, indicating a close relationship among these chemical and biological variables (Guo et al., 2021a). Some research also showed a decreasing trend in EMF colonization and diversity with increasing N availability, while others indicated an increasing abundance of specific EMF taxa in presence of increased soil nutrient contents (Treseder, 2004; Näsholm et al., 2009). Therefore, ambiguity exists in the holistic understanding of EMF regulating factors and community diversity patterns in soil matrix thus generating a need to identify the exact EMF community regulatory factors, especially in the forest ecosystems where plant-mycorrhizal interactions occur at greater rates.

The divergent EMF taxa differ largely in nutrient uptake strategies, the differences are partially represented in the features of their emanating mycelium, which are relevant to the function of nutrient exploitation and transport (Agerer, 2006; Hobbie and Högberg, 2012). Generally, the primary function of the emanating EMF mycelium is to scavenge and transport nutrients from soil matrix to the host plant (Agerer, 2006; Hobbie and Högberg, 2012). However, there are differences reported even in this critical function among different EMF taxa, which means the existence of altering nutrient uptake strategies between the EMF taxa and the host plant species. The EMF communities are characterized by different “exploration types” based on their morphological differences (Agerer, 2006): contact (smooth mantle with few hyphae, close contact with soil substances); short-distance (dense hyphae with short distance); medium-distance (hyphae with intermediary distance, 30–50 mm); and long-distance (few rhizomorphs with long distance) exploration types. Each of the exploration types may represent a distinct foraging strategy under different soil and environmental conditions (Lilleskov et al., 2011). Hence, exploring the patterns of EMF exploration types would highlight EMF responses to environmental changes, and changing EMF functional diversity across different forest ecosystems (Rosinger et al., 2018; Baeza-Guzmán et al., 2022). Nevertheless, little is known about the environmental regulators of EMF functional traits under different forests within the subalpine climatic zone.

The Qinghai–Tibet Plateau is a unique location comprising of different forest systems vulnerable to climate change at regional scale (Han et al., 2021; Wang et al., 2021a,b). Therefore, elucidating the shift of EMF communities and the influencing environmental factors in this Himalayan region is important for understanding future changes in its ecological services. Therefore, in this study, we explored ectomycorrhizal fungal (EMF) community and functional diversity across three different coniferous forests with the following hypotheses:

- The EMF colonization rate, diversity and community structure would differ across three different coniferous forests dominated by different tree species;
- Considering the interactions between above-below ground biotic communities and soil properties, any changes in plant diversity and soil properties will be reflected by variations in EMF community diversity;
- Under varying environmental conditions and plant diversity, the EMF communities will have to adapt and alter nutrients foraging strategy, thereby indicating significant changes in EMF functional traits.

## Materials and methods

### Site description

The study site is in a mountainous region on the Southern Qinghai–Tibet Plateau (28°22′35″–28°25′1″N, 85°24′44″–85°26′49″E). The location is near the Keelung Port, and at an elevation level of 3,000 m a.s.l (meters above sea level). Indian ocean Monsoons impact the weather of the study area, with mean annual precipitation (MAP) and mean annual temperature (MAT) of 635 mm and 3.9°C, respectively; This region experiences long sunlight hours throughout the year (mean annual sunlight duration of 2,639 h per year). The main vegetation at the site is natural secondary subalpine coniferous forest on a dark brown soil (World Reference Base, WRB). From this subalpine coniferous forest zone, three separate coniferous forest sites dominated separately by *Pinus wallichiana*, *Abies spectabilis* and *Picea spinulosa* were selected. The three forest sites were approximately 300 m distance apart. Five forest plots (20 m × 20 m) with a 20 m distance grid, at each of the three forest sites (5 × 3, total of 15 plots) were systematically chosen for soil sampling purposes.

### Root and soil sampling

In each forest plot, soil adhering to fine roots at 0–10 cm depth was collected by shaking and then sieved through > 2 mm ethanol sterilized mesh. These root samplings were done along four different directions and at a distance of ~ 0.5 m from the same tree trunk. The fine root samples were collected from five randomly selected but similar sized dominant trees (~ 30 cm diameter at breast height). Care was taken to trace the fine roots belonged to the same tree. The four fine root and soil samples from each tree were bulked to form one composite sample per tree. Subsequently, the fresh soil and root samples were stored at −20°C and 4°C for soil properties analyses and mycorrhizal morphotyping, respectively.

### Soil properties analysis and plant diversity survey

Soil pH value was determined by a conductivity meter (Mettler-Toledo Instruments, China) in a 1:2.5 soil: water solution. Soil organic carbon (SOC) and total nitrogen (TN) concentrations were measured with a CN-element analyzer (PE2400II, United States; Yang et al., 2021). Ammonium (NH_4_^+^) and nitrate (NO_3_^−^) contents were, respectively, determined by the indophenol blue colorimetric technique and phenol-disulfonic acid colorimetric method (Bao, 2016) after the extraction from the soil samples with 2 mol l^−1^ potassium chloride (KCl) solution. We used the molybdenum blue method after extraction to colorimetrically quantify available phosphorus (AP) from the soil in a 0.5 M sodium bicarbonate (NaHCO_3_) solution at pH 8.5 (Bao, 2016). The total phosphorus (TP) was analyzed colorimetrically (Parkinson and Allen, 1975) after digestion with sulfuric acid-perchloric acid (H_2_SO_4_–HClO_4_).

Plant diversity survey of each forest site plot was conducted in August 2019 by using the method described by Dengler (2009). Different species compositions were recorded in a large plot (20 m × 20 m) for trees, medium-sized plot (3 m × 3 m) for shrubs, and small plot (1 m × 1 m) for herbs. In each plot, alpha-diversity indices (Chao1 index and Shannon index) of plant species were determined by counting the number of individual plants belonging to same species per plot (Pauli et al., 2015).

### Morphotyping

Soil particles and debris of the root samples were carefully and gently brushed using a fine brush and washed under tap water. The tips of fine roots were classified under a dissecting microscope (Nikon SMZ18, Kanagawa, Japan), according to their morphological characteristics such as the shape, color, bifurcation, and the texture of the surface of the mycorrhizas (Agerer, 2001; Pena et al., 2013). The number of root tips, i.e., vital EMF, vital non-EMF, dead roots, and EMF root tips colonized by each EMF morphotype were noted and photographed to record their morphological characteristics. EMF colonization rate was calculated using the following equation:


$$\mathrm{EMF}\;\mathrm{colonizing rate}=\frac{\mathrm{Number of vital}\;\mathrm{EMF}\;\mathrm{root tips}\;}{\mathrm{Number of total root tips}}.$$

For each EMF morphotype, ~ 20 root tips were carefully selected and stored at −20°C until DNA extraction and molecular identification.

### Molecular identification

The total genomic DNA of EMF morphotypes was extracted from the root tips using modified cetyl-trimethylammonium bromide (CTAB) method as described by Roy et al. (2013). This extracted DNA served as template for PCR (Polymerase Chain Reaction) amplification of the fungal internal transcribed spacer (ITS) gene. The ITS1F (5′-TCCGTAGGTGAACCTGCGG-3′) and ITS4R (5′-TCCTCCGCTTATTGATATGC-3′) were used as EMF specific forward and reverse primers in the PCR thermal profile (Yang et al., 2019, 2021). The 25 μl PCR reaction mixture contained: 2 μl template DNA, 1.25 μl of each primer (10 mM), 0.125 μl DNA polymerase (5 μ μl^−1^), 2.5 μl of 10 × Taq buffer, 3 μl MgCl_2_ (23 mM), 0.5 μl of 10 mM dNTP mix and 14.375 μl ddH_2_O. The PCR thermal profile included following steps: initial denaturation at 95°C for 1 min, followed by annealing at 55°C for 30 s and extension for 72°C for 1 min, and a final extension at 72°C for 5 min. The amplified PCR products were purified using Axy Prep DNA Gel Recovery Kit as described by the manufacturers.

The purified PCR products were then used for Sanger sequencing and the sequences were assembled using Staden Package 4.10.^1^ The BLAST analysis against National Center for Biotechnology Information (NCBI)^2^ public sequence databases was conducted, to obtain fungal identification of the sequences with the similarity higher than 98%. All the fungal sequences were deposited in the NCBI GenBank and are available under the accession numbers: MT730591–MT730602 (Supplementary Table S1). Based on the EMF identification from NCBI, the relative abundance of each EMF taxa was calculated as:


$$\begin{array}{l} \mathrm{The relative abundance of each}\;\mathrm{EMF}\;\mathrm{taxa}\\ =\frac{\mathrm{Number of each}\;\mathrm{EMF}\;\mathrm{root tips}\;}{\mathrm{Number of total vital}\;\mathrm{EMF}\;\mathrm{root tips}\;}. \end{array}$$

### Statistical analysis

Prior to the statistical analyses, Shapiro–Wilk and Levene test were used to test the normality and homogeneity of the data. Where not normal, the data were log transformed or non-parametric tests were used for the statistical analysis. Analysis of Variance (ANOVA) was conducted to determine the significant differences in the means of plant diversity indices and soil properties and their influence on the EMF colonization. The EMF alpha-diversity was estimated by richness (Chao1) and diversity (Shannon index). The Pearson’s correlation analyses were used to determine the associations between EMF alpha-diversity and environmental variables (Oksanen et al., 2020).

Redundancy analysis (RDA) were conducted to investigate the impacts of plant diversity and soil properties on EMF communities structure (Yang et al., 2021), and Analysis of Similarities (ANOSIM) was used to calculate the dissimilarities between EMF communities using the Bray-Curtis distance matrix method. Variation Partitioning Analysis (VPA) was used to assess the contributions of each variable to the overall variations in the EMF communities (Liu et al., 2018). All statistical analyses were performed using R statistical software.

## Results

### Plant diversity and soil properties

Across the three forest sites, the plant richness (Chao1 index) remained unchanged (*p* > 0.05; Table 1). However, plant diversity (Shannon index) was higher in forest sites dominated by *P. wallichiana* and *A. spectabilis* in comparison to the *P. spinulosa* dominated forest site (*p* < 0.05; Table 1).

**Table 1:** Soil properties and plant diversity indices across the three studied coniferous forest sites.

Variables	Coniferous forests		
	*Pinus wallichiana*	*Abies spectabilis*	*Picea spinulosa*
Soil properties			
SOC (g kg ^−1^)	40.0 ± 13.1b	134.9 ± 20.7a	106.5 ± 8.1a
TN (g kg^−1^)	2.5 ± 0.7b	8.9 ± 0.1a	6.9 ± 0.0a
TP (mg kg^−1^)	688.4 ± 109.8b	1387.7 ± 116.2a	1329.5 ± 157.3a
NO_3_^−^ (mg kg^−1^)	78.6 ± 18.6b	237.1 ± 55.7a	250.8 ± 49.5a
NH_4_^+^ (mg kg^−1^)	27.4 ± 6.1b	69.9 ± 10.2a	32.7 ± 4.8b
AP (mg kg^−1^)	13.3 ± 2.8a	17.4 ± 3.9a	15.3 ± 2.2a
pH	5.3 ± 0.2a	4.9 ± 0.2a	5.0 ± 0.1a
Plant diversity			
PR	33.0 ± 2.0a	34.0 ± 1.0a	30.0 ± 1.0a
PS	3.1 ± 0.1a	3.2 ± 0.2a	2.9 ± 0.2b

SOC, soil organic carbon; TN, total nitrogen; TP, total phosphorus; NO_3_^−^, nitrate nitrogen; NH_4_^+^, ammonia nitrogen; AP, available phosphorus; PS, plant Shannon index; PR, plant Chao1 richness index. Values indicate means ± standard errors (SE; *n* = 5). Different letters indicate significant differences between means (*p* < 0.05).

Among the measured soil chemical properties, soil organic carbon (SOC), total nitrogen (TN), soil nitrate (NO_3_^−^) and total phosphorus (TP) had lower contents in the *P. wallichiana* forest soils than the contents in *A. spectabilis* and *P. spinulosa* dominated forest soils (*P*_SOC_ = 0.02, *P*_TN_ = 0.01, *P*_NO3‑_ = 0.031, *P*_TP_ = 0.05). The highest values for these soil contents were found in forest site where *P. spinulosa* were abundant (SOC: 177.18 g kg^−1^; TN: 7.76 g kg^−1^; TP: 1735.50 mg kg^−1^; Table 1). The Ammonium (NH_4_^+^) contents were significantly higher (*p* = 0.02) and twice the amount in the *A. spectabilis* forests (108.32 mg kg^−1^), than the *P. wallichiana* and *P. spinulosa* forest sites. Except for nitrate N (NO_3_^−^) contents, the amounts of all other soil parameters were significantly higher in the *A. spectabilis* dominated forests than the other two forests and showed a trend of *A. spectabilis* > *P. spinulosa* > *P. wallichiana*. The soil pH and available phosphorus (AP) did not significantly differ among three forest soils (*P*_pH_ = 0.195, *P*_AP_ = 0.586; Table 1).

### Ectomycorrhizal fungal (EMF) colonization rate and alpha-diversity

**Figure 1:**
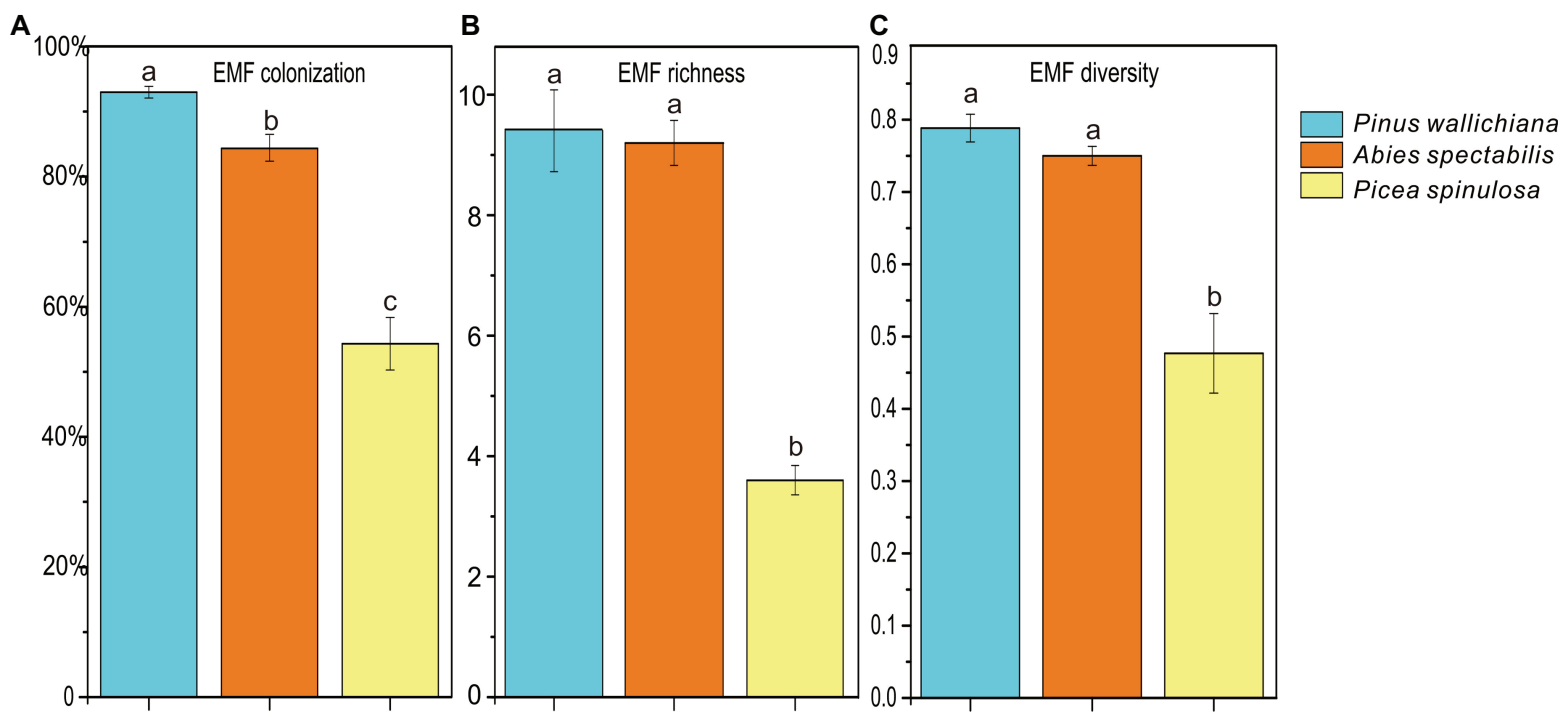
Ectomycorrhizal fungal (EMF) colonization **(A)** and alpha-diversity indices **(B, C)** across three coniferous forests. Different letters indicate significant differences at *p* < 0.05 (ANOVA) between means (*n* = 5).

The EMF colonization rate and alpha-diversity indices varied highly among the three forests with EMF colonization rate differing significantly (*p* < 0.001; Figure 1A). The lowest EMF colonization rate was in the *P. spinulosa* forest (42.2%), while the highest rate was observed in the *P. wallichiana* forests (95.1%). The EMF richness (Chao1 index) ranged from 3 to 10 and was significantly lower in the *P. spinulosa* (3.6) than in the *P. wallichiana* (9.4) and *A. spectabilis* forests (9.2; Figure 1B; *p* = 0.012). The EMF diversity (Shannon index) of *P. spinulosa* forest ranged between 0.259 to 0.543, and was significantly lower than *P. wallichiana* (0.789) and *A. spectabilis* (0.749) forests (*p* < 0.05; Figure 1C). The Pearson correlations analysis considering the soil properties and the EMF community characteristics at the three forests sites showed significant negative relations between soil NO_3_^−^ and TP and, all EMF characteristics such as the EMF colonization rate and EMF alpha-diversity indices (*p* < 0.05) except for NO_3_^−^ and EMF richness, which had no significant negative correlationship (Table 2).

**Table 2:** The Pearson correlations considering the soil properties and the ectomycorrhizal fungal (EMF) community properties at all the three forest sites (*Pinus wallichiana*, *Abies spectabilis*, and *Picea spinulosa*).

	EMF properties		
Variables	Colonization	Richness	Diversity
pH	0.142	0.273	0.383
SOC	−0.429	−0.209	−0.306
TN	−0.473	−0.303	−0.417
NH_4_^+^	−0.003	0.323	0.174
NO_3_^−^	−0.541^*^	−0.459	−0.519^*^
TP	−0.553^*^	−0.551^*^	−0.647^**^
AP	−0.030	−0.054	−0.023
PR	0.099	0.249	0.301
PS	0.323	0.297	0.359

* Significant correlations at the level of *p* < 0.05.

** Significant correlations at the level of *p* < 0.01.

Values indicate *r* of the soil properties and the ectomycorrhizal fungal (EMF) community properties. Bold values indicate significant correlations (*p* < 0.05).

### Variation in EMF taxonomic community composition

**Figure 2:**
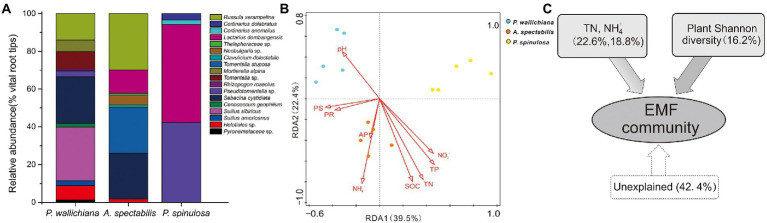
The community composition **(A)**, redundancy analysis **(B)** and variation partition analysis **(C)** of ectomycorrhizal fungal community across the three forests (*Pinus wallichiana*, *Abies spectabilis*, and *Picea spinulosa*). TN, total nitrogen; NH_4_^+^, ammonia nitrogen.

The EMF taxonomic community compositions varied strongly within in the three forests and differed significantly as indicated by ANOSIM analysis (*p* < 0.05; Figure 2A; Supplementary Table S1). The relative abundance of EMF species *Suillus sibiricus* (24.99%) and *Sebacina cystidiata* (24.75%) were highest in *P. wallichiana* forest, followed by *Russula xerampelina* (15.23%), *Tomentella* sp. (8.29%), while *S. cystidiata* (25.04%), *R. xerampelina* (27.67%) and *Tomentella stuposa* (22.41%) were dominant in *A. spectabilis* forest. The main dominant species of the *P. spinulosa* forest were *Lactarius dombangensis* (51.93%) and *Pseudotomentella* sp. (42.26%; Figure 2A; Supplementary Table S1).

The results of Redundancy Analysis (RDA) indicated soil TN, NH_4_^+^ and plant diversity significantly affect the EMF taxonomic community structures across the studied three forests (Figure 2B; Supplementary Tables S2 and S3). Whereas the Variation Partitioning Analysis (VPA) showed, soil TN and NH_4_^+^ explained 22.6 and 18.8% of the total variations in EMF community structure, respectively. The plant diversity on the other hand explained only 16.2% of the total variations in EMF community structure within the three forests (Figure 2C; Supplementary Table S2).

**Figure 3:**
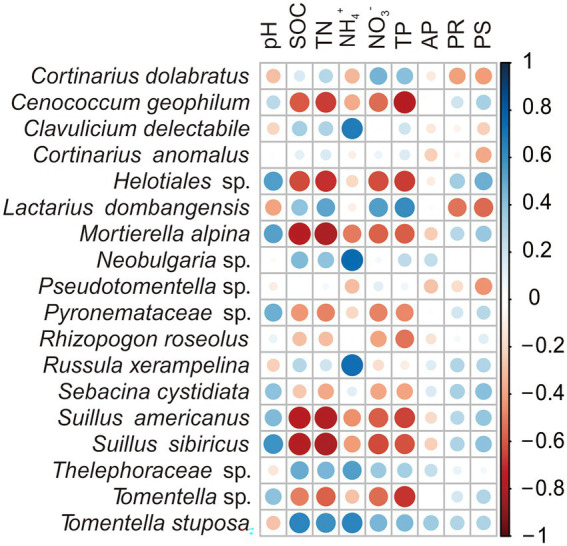
Pearson correlations between ectomycorrhizal fungi (EMF) species and environmental factors across the three forests in the study area. Values indicate means ± standard errors (SE; *n* = 5).

The Pearson correlations analysis between each EMF taxa and different environmental variables showed significant positive relation between soil pH and increased relative abundance of fungal species *Helotiales* sp., *S. sibiricus* and *Mortierella alpina* (*p* < 0.05). The soil TN and SOC negatively correlated with decreased relative abundance of *Helotiales* sp., *S. americanus*, *S. sibiricus*, *C. geophilum* and *M. alpine* (*p* < 0.05), but had significant positive relationship with the relative abundance of *T. stuposa* (*p* < 0.05). Likewise, NO_3_^−^ and TP negatively correlated with the relative abundance of *Helotiales* sp., *S. americanus*, *S. sibiricus*, *Tomentella* sp. and *M. alpina*, but had positive relations with relative abundance of *L. dombangensis*. The NH_4_^+^ also had significant positive relation with the relative abundance of *T. stuposa*, *C. delectabile*, *Neobulgaria* sp., *Thelephoraceae* sp. and *R. xerampelina* (*p* < 0.05). While, plant diversity was significantly negatively related with the relative abundance of *L. dombangensis* (*p* < 0.05; Figure 3; Supplementary Table S4).

### Variation in EMF exploration types

Changes in EMF taxonomic structures resulted in corresponding changes in soil exploration types. The soil exploration types of EMF communities differed across the three forests; four EMF exploration types (contact, short-distance, medium-distance and long-distance) were identified in total.

**Figure 4:**
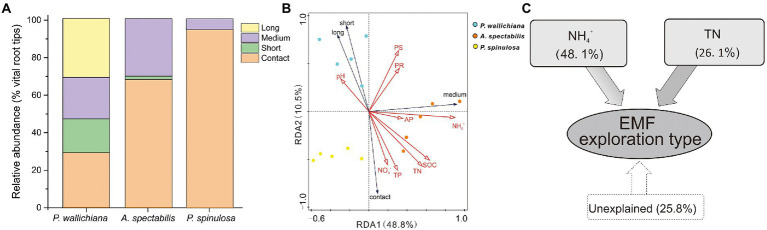
Exploration types mode-based community structure **(A)**, redundancy analysis **(B)** and variation partition analysis **(C)** of ectomycorrhizal (EMF) species across the three forests (*Pinus wallichiana*, *Abies spectabilis*, and *Picea spinulosa*). TN, total nitrogen; NH_4_^+^, ammonia nitrogen.

The relative abundance of contact exploration type was significantly lower in *P. wallichiana* forests (relative abundance: 28.90%) than in the *A. spectabilis* (67.76%) and *P. spinulosa* abundant forest sites (94.19%; *p* < 0.05; Figure 4A). No long-distance exploration types were detected in *A. spectabilis* and *P. spinulosa* forests, compared to the relative abundance of 31.27% in *P. wallichiana* forests (Figure 4A).

Based on ANOSIM analysis, the soil exploration types of EMF communities differed significantly across the three forests (Supplementary Table S5). RDA analysis showed significant influences of soil NH_4_^+^ and TN on EMF community structures when considering the soil exploration types (*P*_NH4+_ = 0.002, *P*_TN_ = 0.036. Figure 4B; Supplementary Table S6). The soil NH_4_^+^ explained 48.1% and TN explained 26.1% of the total variations in EMF exploration types in the three forest types, respectively (Figure 4C).

## Discussion

### Soil nutrients influence EMF colonization rate and alpha-diversity

Soil nutrient cycling in forest ecosystems is largely based on the ecological functioning of ectomycorrhizal fungi (EMF), nearly 75% of phosphorus (P) and 80% of nitrogen (N) acquisition seems to be facilitated by mycorrhizal fungi (Hobbie and Hobbie, 2006; van der Heijden et al., 2008). Compared to the soils of boreal forest ecosystems dominated by Norway spruce (*Picea abies*; Kjøller et al., 2012; Almeida et al., 2019), the soils in our study had lower amounts of nitrogen (TN) and available phosphorus (AP) in presence of acidic pH (soil pH ~5). Such lower pH accounts for N and P limitation in soils (Härdtle et al., 2004; Stark et al., 2014), especially the N status that influenced EMF community composition (Kjøller et al., 2012). Further, in coniferous forests characterized as shown in earlier study by low nutrient soils, the diversity in EMF composition affected the decomposition of aromatic and lignified substrates that were common to coniferous and deciduous litter (Bardgett and McAlister, 1999; Six et al., 2006). A few studies showed positive relationships between higher EMF richness and diversity under increased P uptake efficiency in European beech (*Fagus sylvatica*) forest ecosystems (Köhler et al., 2018) and N acquisition in boreal forests (Helmisaari et al., 2009). Albeit the studies were conducted at sapling levels and such observations were not reported in forest ecosystems dominated by adult trees.

Furthermore, the EMF are host specific and have high species diversity and assemblage which may vary across habitat and spatial scales (seedling and root tip scales; Rosinger et al., 2018; Yoo et al., 2022) and may be further escalated with changes in plant species across different habitats. Therefore, in agreement with our first hypothesis, the EMF colonization rate and EMF diversity differed across three coniferous forests dominated by different tree species. The EMF were least diverse and showed decreased colonization rates in forest ecosystems with abundant *P. spinulosa* and higher soil nutrient contents. The nitrate and phosphorous contents in soil were the primary indicators of EMF variability in our study as seen by RDA analysis. This is in line with previous studies showing soil environmental variables (e.g., the soil N, soil N:P ratio and soil pH) having significant influence on EMF diversity at local scale (van der Linde et al., 2018).

The nutritional mutualisms between EMF and plants is beneficiary for both participants and bridge soil nutrients and plant photosynthetic C pools, especially in forest ecosystems where available soil nutrients are limited (Johnson and Graham, 2013). Plants usually allocate 20–40% of C to EMF, which in turn forage soil nutrients that are not easily accessible to plants, meaning that host plants depend on fungal partner for resource acquisition. In a different scenario, the host plant dependence on EMF colonization may be reduced under increased soil nutrients as indicated by the reduced plant C allocation thus affecting the EMF colonization rate and diversity (Wyatt et al., 2014). In our study, EMF colonization rate and diversity negatively correlated with soil TP and NO_3_^−^, which was similar to previous studies (Cline et al., 2018; Rosinger et al., 2018; Yang et al., 2021). Such negative correlation between NO_3_^−^ and EMF colonization rate and diversity illustrated the diverse effects of N limitation.

In other studies, EMF communities were shown to be highly sensitive towards any changes in N than P availability in soils (Almeida et al., 2019). While also, the EMF diversity declined in presence of higher TN in the organic layer and remained unaffected to changes in the soil TP levels which only lead to the changes in EMF taxonomic community structure (Zavišić et al., 2016). On the other hand, Teste et al. (2016) indicated decreasing EMF hyphae with low P availability, perhaps indicating a divergent assembly mechanism under forests with different nutrients status (Lang et al., 2016). Moreover, the N:P ratio stoichiometry may also regulate the variations in soil mycorrhizal community diversity and not just the changes in single element (Johnson, 2010).

No changes in EMF alpha diversity were seen despite the changes in plant diversity across the three forest sites. This contradicts our second hypothesis based on findings of earlier studies that have demonstrated positive relationship between host plant and EMF community diversity (Dickie, 2007). The unaffected EMF alpha diversity can be attributed to the case that the majority of EMF detected could be conifer associates while also a large proportion can be non-host specific mycorrhizal communities. In this work, EMF were studied at root tip scale where they may be less segregated and that the early colonizing species may have monopolized root tips at the expense of late colonizers (Helmisaari et al., 2009). Nevertheless, several studies have also shown no direct correlation between plant and EMF diversity (Wardle, 2006). Moreover, the diversity of EMF community was more regulated by different environmental factors and/or was influenced by environmental filtering (Orwin et al., 2010).

### Soil nitrogen and plant diversity affect EMF community structure and functional traits

Although plant and EMF diversity did not correlate, the results of RDA analysis in this study yet represented plant diversity and soil properties as local aspects that determine the variations in EMF taxonomic community structure in the three subalpine forest sites. The earlier studies have reported higher plant diversity induced changes in EMF communities: (i) by directly altering the EMF community composition through changes in the quantity of photosynthetic C supplied to the symbiotic partner (Gao and Guo, 2014), and (ii) by indirectly creating specific niche environment in soil matrix through differential litter mixture and root exudates inputs (Pei et al., 2016; Yang et al., 2019). In other circumstances, plants also manifested EMF colonization by harboring more diverse or specialist mycorrhizal communities within the same habitat to decompose C sources such as the lignin, cellulose, etc. (Berg, 2000; Lindahl et al., 2007; Allison et al., 2013). Likewise, in this study as well, a total of 16.2% variations in EMF taxonomic community structure was observed due to changes in plant diversity, thereby illustrating the marked influence of the latter on EMF dynamics in forest ecosystems. The plant diversity and the relative abundance of EMF community in *P. spinulosa* forest were negatively correlated in this study, which may be due to the already lower number of EMF species in the investigated site. While also it can be attributed to the selective filtering of mycorrhizal communities based on the evolutionary history of the fungi-host association (Roy et al., 2013). Similar findings were reported by Courty et al. (2007) where *Lactarius quietus* were associated with oak and *Suillus* sp. with Pinaceae (Zhang et al., 2022). In other study, some common EMF (*Cenococcum geophilum*) depicted wide host range (Yang et al., 2019). Accordingly, co-evolution between host plants and associated EMF cannot be neglected as a potential reason towards the observed EMF specificity in our study (Rochet et al., 2011), which may determine the functional traits of the existing EMF communities in the three studied forest ecosystems.

In addition to plant diversity, the local soil properties in the studied forest soils also control the EMF taxonomic community composition. Among these soil variables, soil nitrogen and it’s form such as the ammonium (NH_4_^+^) and total nitrogen (TN) were the key regulators in our study, and this is in line with the results of previous studies displaying close relationship between soil NH_4_^+^ and EMF community composition in forest soil systems (Suz et al., 2017; Baeza-Guzmán et al., 2022). Different EMF taxa respond differently to the varying N availability. This study indicated that *Helotiales* sp., *Suillus americanus*, *Suillus sibiricus*, *Cenococcum geophilum* were negatively correlated with higher TN. The similar result was observed by Yang et al. (2021) as the EMF taxa: *Cenococcum geophilum*, and *Helotiales* sp. were negatively correlated with increased N availability. Whereas *Tomentella stuposa* and *Russula xerampelina* preferred relatively high soil N conditions (Cox et al., 2010; Kjøller et al., 2012), and had higher abundance in *A. spectabilis* and *P. spinulosa* forests in our study. A probable explanation for this occurrence can be that even different EMF species within the same genus may respond both positively or negatively to one variable at the same time; for example, within the *Lactarius* genus, *Lactarius rufus* and *Lactarius hepaticus* were negative and positive indicators for soil TN, respectively (van der Linde et al., 2018). Further, Morrison et al. (2016) have also shown that most *Russula* species were inexpedient under N-enriched condition except EMF *Russula vinacea*. This inconsistency may be attributed to more extensive role of other soil and/or environmental factors that govern EMF taxonomic community composition and structure, which needs further investigations.

Alterations in EMF taxonomic structures resulted in corresponding changes in soil exploration types across the studied three forests. The distinction of EMF exploration types may help to elucidate the divergent response of EMF communities to environmental variables, with respect to their ecophysiological effectivity (Agerer, 2006). In this study, functional diversity in the EMF community was least where soil N availability was higher. A more diverse EMF with foraging mycelium were observed in *P. wallichiana* forests in presence of lower soil N contents. This indicated the sensitivity and adaptability of EMF mycelium to shift their functional role depending on soil nutrients status and availability. A similar result was reported in a recent study conducted in high altitude regions with poor nutrients and harsh environment (Baeza-Guzmán et al., 2022). The same study showed that the EMF communities developed abundant mycelium in order to acquire more nutrients for the host plant.

EMF can mineralize organic forms of N and P by releasing hydrolytic and oxidative enzymes, while also can effectively forage nutrients from inorganic sources (e.g., NH_4_^+^, NO_3_^−^, phosphate) through their hyphal network (Smith and Read, 2008). Diverse EMF exploration types have greater implications for improved ecosystem functioning. Long-distance types often showed higher nutrient uptake efficiency (Koide et al., 2014) and facilitated organic N acquisition (Hobbie and Agerer, 2010; Lilleskov et al., 2011; Tedersoo and Smith, 2013). Whereas, short-range exploration types preferentially foraged nitrate and ammonium (Cox et al., 2010; Hobbie and Agerer, 2010; Tedersoo and Smith, 2013). In this study, we observed increases of contact exploration foraging types in *A. spectabilis* (relative abundance: 67.76%) and *P. spinulosa* (94.19%) forests, than *P. wallichiana* (28.90%) forests where soil TN contents were comparatively low. No long-distance exploration types were detected in *A. spectabilis* and *P. spinulosa* forests, compared to the relative abundance of 31.27% in *P. wallichiana* forests. This is in line with the recent study (Guo et al., 2021b), showing an increase in soil N availability resulted in increase in abundance of EMF genera belonging to contact, short-distance exploration types in comparison to the decreased medium-distance (e.g., *Tomentella* sp.) and long-distance (e.g., *Suillus* sp., *Inocybe* sp.) EMF exploration types.

On the other hand, due to the different amount of emanating hyphae, contact exploration type require less C than the long-distance EMF exploration type (Fransson, 2012). Recently, Yang et al. (2021) reported that the soil mycelium foraging EMF communities shift from the high- to low- biomass type under high levels of N contents in soil. This means that plants have more labile N under higher soil N availability. This greatly reduces the reliance of plant hosts on mycelial EMF, especially in the events of long-distance transport of N resources. This may also lower the allocation of C to EMF symbionts by the host plants. Indeed, more work is needed for an explicit explanation of exploration types observed in this study. This reduced host plants allocation of C to EMF symbionts for long-distance transport of N resources. A connection of this explanation with the exploration type observed in this study is needed.

## Conclusion

This study explored how ectomycorrhizal community and functional diversity respond to varying soil chemical properties and plant diversity across three different coniferous forests (*Pinus wallichiana*, *Abies spectabilis*, *Picea spinulosa*). The EMF community and functional diversity differed across the three forest sites. The soil total phosphorus (TP) and nitrate (NO_3_^−^) contents were key predictors of EMF colonization and EMF Shannon diversity index. While soil TP controlled EMF richness. Additionally, the EMF taxonomic community structures were explained by soil total nitrogen (TN), ammonium (NH_4_^+^) and plant diversity, while NH_4_^+^ and TN in soils were the local causes for the differences observed in exploration types of EMF communities in this study. The findings of this work advocate the complex interactions between above and below ground biotic communities especially the EMF and soil properties. However, the inferences were only related to soil chemical characteristics influencing root tip scale EMF colonization rate and diversity. Therefore, further work on understanding the effects of other abiotic and biotic variables such as the soil moisture, plant trait and plant productivity on EMF fungal trophic guilds is needed. Regardless, the results contribute to comprehensive understanding of EMF community diversity and characteristic and local causes determining their regulation in subalpine coniferous forests dominated by different coniferous tree species.

## Data availability statement

The datasets presented in this study can be found in online repositories. The names of the repository/repositories and accession number(s) can be found in the article/Supplementary material.

## Author contributions

NY: investigation, conceptualization, visualization, formal analysis, writing—original draft, and writing—review and editing. JH: investigation, visualization, formal analysis, and writing—original draft. JZ, YZ, and XL: investigation and methodology. PB and DL: writing—review and editing. HR: resources, funding acquisition, validation, and project administration. WX and LM: conceptualization, funding acquisition, visualization, supervision, and writing—review and editing. All authors contributed to the article and approved the submitted version.

## Funding

The study was support by the following programs: Strategic Priority Research Program of the Chinese Academy of Sciences (no. XDB31000000), National Key Research and Development Program of China (no. 2021YFD2200403), and Natural Science Foundation of China (no. 31870506, 41907029, and 32071594).

## Conflict of interest

The authors declare that the research was conducted in the absence of any commercial or financial relationships that could be construed as a potential conflict of interest.

## Publisher’s note

All claims expressed in this article are solely those of the authors and do not necessarily represent those of their affiliated organizations, or those of the publisher, the editors and the reviewers. Any product that may be evaluated in this article, or claim that may be made by its manufacturer, is not guaranteed or endorsed by the publisher.

## Footnotes

^1^ http://staden.sourceforge.net

^2^ http://www.ncbi.nlm.nih.gov/
